# Functional Characterization of Tachykinin in Regulating Feeding and Energy Metabolism in the Chinese Oak Silkworm, *Antheraea pernyi*

**DOI:** 10.3390/insects17030257

**Published:** 2026-02-28

**Authors:** Guobao Wang, Yunhan Zhang, Yong Wang

**Affiliations:** 1 College of Biology and Oceanography, Weifang University, Weifang 261061, China; gbwang0216@163.com (G.W.); 19053637585@163.com (Y.Z.); 2 Insect Resource Innovation Center for Professional Technology of Liaoning Province, Shenyang 110866, China; 3 College of Bioscience and Biotechnology, Shenyang Agricultural University, No. 120 Dongling Road, Shenyang 110866, China

**Keywords:** *Antheraea pernyi*, tachykinin, feeding, energy metabolism, RNA interference

## Abstract

Tachykinins (TKs), a conserved family of neuropeptides, play critical roles in regulating multiple physiological processes such as feeding and energy metabolism in insects. This study identified the TK gene (*ApTK*) from the Chinese oak silkworm, *Antheraea pernyi*, an economically important insect species. Bioinformatic analysis showed that ApTK possesses four FX_1_GX_2_R motifs (X_1_ and X_2_ represent variable amino acid residues), comprising FMGVR, FYGVR, FIGVR, and FFGMR, in the C-terminus and shares a close phylogenetic relationship with TKs from *Bombyx mori* and *Manduca sexta*. Tissue-specific expression profiling demonstrated that *ApTK* was mainly expressed in the brain and midgut. Starvation–refeeding experiments showed that the expression of *ApTK* was significantly upregulated during food deprivation and returned to baseline after refeeding, evincing its involvement in hunger signaling. RNA interference (RNAi)-mediated knockdown of *ApTK* led to a significant increase in larval body weight and increased levels of triglyceride, glycogen, and trehalose, indicating enhanced energy storage. Collectively, these results demonstrate that *ApTK* acts as a key regulator in restraining feeding and modulating energy homeostasis in *A. pernyi*. Our findings provide insights into the neuroendocrine mechanisms underlying feeding behavior and energy metabolism in *A. pernyi*.

## Simple Summary

This study identified and characterized the tachykinin gene (*ApTK*) in the Chinese oak silkworm, *Antheraea pernyi*. *ApTK* encodes a peptide with four FX_1_GX_2_R motifs, where X_1_ and X_2_ represent variable amino acid residues, and is most highly expressed in the brain and midgut. Our results showed that *ApTK* expression increases during starvation and decreases after refeeding, indicating it responds to hunger. When *ApTK* was silenced using RNA interference, larvae gained more weight and had higher levels of triglyceride, glycogen, and trehalose. These findings demonstrate that *ApTK* acts to restrain feeding and limit energy storage, playing a key role in regulating energy balance in *A. pernyi*.

## 1. Introduction

Neuropeptides, as a class of peptide compounds secreted by neurosecretory cells, can act as neurohormones, neurotransmitters, and neuromodulators and modulate diverse behaviors and physiological processes, such as growth, development, reproduction, metabolism, circadian rhythm, metamorphosis, and stress tolerance [1,2]. Tachykinins (TKs) are one of the largest families of neuropeptides across vertebrates and invertebrates [3]. TKs are a group of conserved and multifunctional neuropeptides present throughout the bilaterians, with FX_1_GX_2_R–amide carboxy termini, where X_1_ and X_2_ represent variable amino acid residues, in arthropods including insects [4], and they play an essential role in neural transmission or as neuroregulatory substances in the central and peripheral nervous systems [5,6,7]. Insect TKs are mainly expressed in the central nervous system and intestine [8] and are shown to be involved in the synthesis and perception of hormones and modulation of the excretory system [9].

Recently, TKs have been found to regulate a series of feeding processes including food searching and intake, digestion, absorption, and energy metabolism. In *Drosophila melanogaster*, the sensitivity of the larvae to odorants is reduced following the disruption of TK signaling using RNA interference (RNAi) [10]. The insulin signaling pathways are strictly regulated by TK signaling in the brain and midgut of the fruit fly; they both participate in maintaining the level of trehalose, resistance to starvation, and response to oxidative stress [11,12]. In *Bombyx mori*, upregulation of TKs could reduce the larval body weight and significantly shorten the time taken for the larvae to search for food after starvation [13]. In the blood-feeding bug *Rhodnius prolixus*, TKs significantly increase the frequency and amplitude of peristaltic contractions of the salivary glands and hindgut [14]. Nutrients can upregulate the secretion of TKs by midgut endocrine cells; affect the activities of digestive enzymes such as amylases, proteases, and lipases; and regulate intestinal lipid metabolism in the cockroach *Periplaneta americana* and *D. melanogaster* [5,15].

The Chinese oak silkworm, *Antheraea pernyi,* is a typical lepidopteran insect species and a rising model organism due to a variety of advantages such as its short period of reproduction, larger body size facilitating experimental manipulation, and clear genetic background [16]; it is also an economically important insect used for silk production and food resource [17]. *A. pernyi* is an undomesticated wild insect species. Environmental factors such as temperature, humidity, or potential pathogens can affect the feeding behavior, thus influencing the yield and quality of pupae or cocoon silk [18]. Based on the crucial roles of TKs in regulating feeding behavior in insects, this study revealed the regulatory mechanism of the TK signaling system in feeding and energy metabolism processes of *A. pernyi* by analyzing the sequence characteristics, phylogenetics, and transcript expression of the TK gene and applying RNAi for functional verification. The results advance understanding of the molecular mechanisms underlying the regulation of feeding behavior in lepidopteran insects.

## 2. Materials and Methods

### 2.1. Insect Rearing

The *A*. *pernyi* strain Xuanda was used in this study. Larvae were reared on fresh leaves of *Quercus wutaishanica* under natural conditions. The rearing temperature was maintained at 25 ± 2 °C with relative humidity of 60 ± 10% and a photoperiod of 14 L:10 D [19].

### 2.2. Identification and Bioinformatic Analysis of A. pernyi Tachykinin Gene (ApTK)

The total RNA of the *A*. *pernyi* larvae was extracted using an RNAiso Plus kit following the manufacturer’s instructions (Takara, Tokyo, Japan), and the quality and concentration were identified by NanoDrop spectrophotometry (Thermo, Waltham, MA, USA). A sample (2 μg) of total RNA was used as a template to synthesize cDNA using the GoScript^TM^ Reverse Transcription System (Promega, Madison, WI, USA) according to the protocol. The open reading frame (ORF) sequence of *ApTK* was obtained from the transcriptomic database of *A. pernyi* [20]. RT-PCR was applied to validate the accuracy of the sequence with the primers in Table S1. The amino acid sequence of ApTK was deduced based on the ORF. The signal peptide was predicted by SignaIP 6.0 (https://services.healthtech.dtu.dk/service.php?SignalP, accessed on 28 January 2026). Domain structures were predicted using the SMART program (https://smart.embl.de/, accessed on 28 January 2026). Phosphorylation sites were predicted using NetPhos 3.1 (https://services.healthtech.dtu.dk/service.php?NetPhos-3.1, accessed on 28 January 2026). Intrinsically disordered regions (IDRs) were predicted using UCL-CS Bioinformatics Introduction (https://bioinf.cs.ucl.ac.uk/, accessed on 28 January 2026). Multiple amino acid sequence alignment was conducted using ClustalX 2.0 (http://www.clustal.org/clustal2/, accessed on 28 January 2026). The results were visualized using GeneDoc 2.7 (https://genedoc.software.informer.com/, accessed on 28 January 2026). MEGA 7.0 was used to construct the phylogenetic tree using the neighbor-joining method under *n* = 1000 bootstrap replicates [21].

### 2.3. Analysis of the Expression of ApTK in Different Tissues of A. pernyi Larvae

Tissues including the brain, silk gland, midgut, Malpighian tubules, fat body, epidermis, and hemocytes were dissected from fifth-instar larvae on day 1. RNA extraction from each tissue and subsequent synthesis of cDNA were performed using the kits as described above. Then RT-PCR reactions were conducted on a T100 thermal cycler (Bio-Rad, Hercules, CA, USA) using the following procedure: 94 °C for 3 min, followed by 30 cycles of denaturation at 95 °C for 15 s, annealing at 60 °C for 15 s, and 15 s extension at 72 °C. The housekeeping gene *Actin1* was used as the internal reference gene (GenBank accession number: KC242321.1) [20]. The primer sequences are provided in Table S1. The PCR products were detected using agarose gel electrophoresis.

Quantitative RT-PCR (qRT-PCR) was also used to analyze the expression pattern of ApTK in different tissues. According to the instructions for the SYBR^®^ Premix Ex Taq^TM^ Kit (TaKaRa, Tokyo, Japan), all reactions were run in triplicate on an ABI7300 real-time PCR system (Ambion, Austin, TX, USA) using a 20 μL reaction volume with the procedure of 40 cycles of 94 °C for 3 s and 60 °C for 31 s, followed by amplified product dissociation for specificity assessment. The relative transcript level of each gene was calculated using the 2^−ΔΔ*C*_T_^ method using the *C*_T_ values obtained from the reactions [22]. The expression of *Actin-1* was used to normalize that of the target gene. The primers specific to the examined gene are listed in Table S1.

### 2.4. Starvation–Refeeding Treatment for A. pernyi Larvae

Newly molted fifth-instar larvae with uniform growth were separated into control and treatment groups with 40 larvae in each group. The control group was maintained on normal feeding throughout the experiment, while the treatment group was subjected to starvation and refeeding thereafter. Five larvae were collected from each group at each time point (24 h, 48 h, and 72 h) for subsequent experiments. Total RNA was then extracted from the whole body of each larva in the control and treatment groups and reverse-transcribed into cDNA. The expression profile of *ApTK* was subsequently analyzed by qRT-PCR analysis.

### 2.5. RNAi for ApTK

Small interfering RNA (siRNA) targeting *ApTK* was designed as the following sequence: 5′-GAUUAUACAUCGAACGAAATT-3′ (forward) and 5′-UUUCGUUCGAUGUAUAAUCTT-3′ (reverse). It was then synthesized by Sangon Biotech (Shanghai, China). Newly molted fifth-instar larvae with uniform growth were separated into three groups. For RNAi experiments, the larvae were firstly anesthetized on ice for 20 min [23]. Then, 1 μL of siRNA solution (1.5 nmol/μL, approximately 30 ng per larva) was injected into the cuticle between the 9th and 10th somites of each newly molted fifth-instar larva with a microinjector (Hamilton, Bonaduz, Switzerland) [24]. Larvae without injection and injected with DEPC water of the same quantity were set as the control and mock injection groups, respectively. Each group consisted of 90 larvae. All larvae were reared under normal conditions post-injection. Total RNA was extracted from the brain and midgut in the control, mock, and siRNA groups at 24 h, 48 h, and 72 h post-RNAi, and qRT-PCR was used to evaluate the silencing efficiency after injection of siRNA for *ApTK*. The body weight of the larvae in all groups was measured at 24 h, 48 h, and 72 h post-RNAi.

### 2.6. Measurement of Glycogen, Trehalose, and Triglyceride Contents in A. pernyi Larvae

The *A. pernyi* larvae from the control, mock, and siRNA groups at 24 h, 48 h, and 72 h post-RNAi were used for quantifying glycogen, trehalose, and triglyceride according to the instructions for glycogen, trehalose, and triglyceride content assay kits (Sangon Biotech, Shanghai, China), respectively. The whole body of each larva was homogenized in cold PBS on ice using a homogenizer. The homogenate was centrifuged at 10,000× *g* for 15 min at 4 °C. The resulting supernatant was collected for subsequent assays.

The glycogen content was determined using the anthrone–sulfuric acid method. Briefly, an aliquot of the supernatant was digested with 30% KOH, and glycogen was precipitated with ethanol. The pellet was resuspended in distilled water and reacted with anthrone reagent. After boiling and cooling, the absorbance was measured at 620 nm. The glycogen concentration was calculated using a glycogen standard curve. For the determination of the trehalose content, the supernatant was incubated with trehalase to hydrolyze trehalose into glucose. The generated glucose was then quantified using a glucose oxidase/peroxidase reagent. Absorbance was read at 510 nm. The trehalose concentration was determined from a standard curve. The triglyceride content was determined based on the glycerol-3-phosphate oxidase-peroxidase (GPO-POD) method. The supernatant was mixed with the working reagent containing lipase, glycerol kinase, and GPO-POD, then incubated at 37 °C for 10 min; the absorbance was measured at 550 nm. A triolein standard was used for calibration.

### 2.7. Statistical Analysis

Data from biological replicates in each group are presented as means ± standard deviations. Comparisons among multiple groups were performed using ANOVA. The levels of statistically significant difference were set at * *p* < 0.05, ** *p* < 0.01, and *** *p* < 0.001. The graphs were generated using GraphPad Prism 6.0 software (San Diego, CA, USA).

## 3. Results

### 3.1. Sequence Characteristics and Tissue Distribution of ApTK

**Figure 1:**
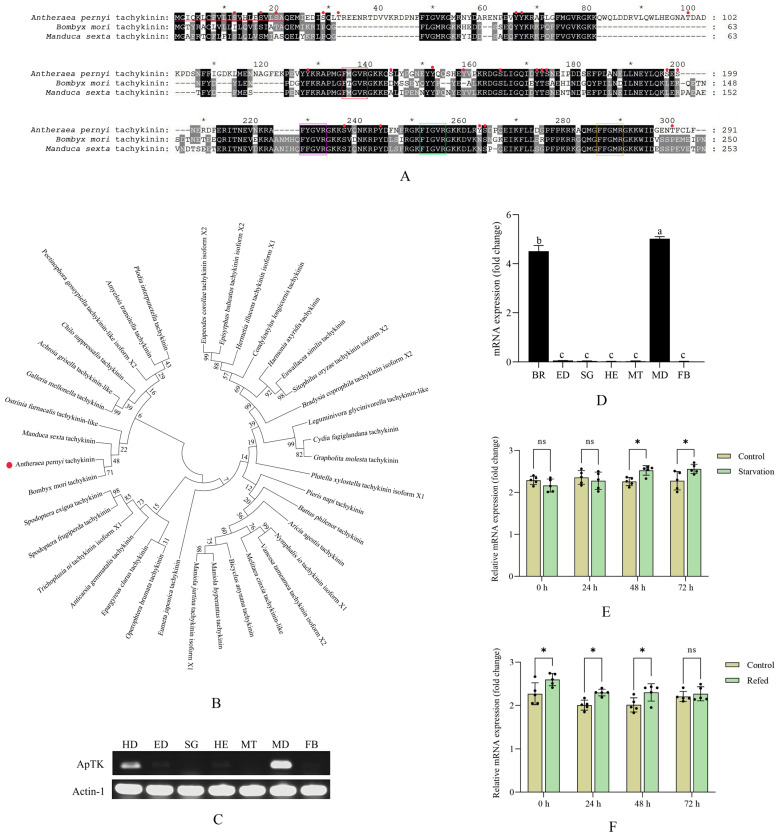
Bioinformatics and expression pattern analysis of ApTK. (**A**) Amino acid sequence alignment of ApTK and TKs from other lepidopteran insects. The red horizontal line indicates the predicted signal peptide sequence, red dots represent predicted phosphorylation sites, and the FX_1_GX_2_R motifs are highlighted by boxes in different colors. “*” indicates every twenty amino acids. For example, the first “*” represents the 10th amino acid, the second “*” represents the 30th amino acid, and so on. (**B**) Construction of the phylogenetic tree for TKs from *A. pernyi* and other lepidopteran species. Detailed information on TKs from the lepidopteran insects in (**A**,**B**) used for amino acid sequence alignment and phylogenetic analysis is shown in Table S2. (**C**) Tissue expression analysis of *ApTK* in *A. pernyi* larvae on the third day of the fifth instar by RT-PCR. (**D**) qRT-PCR analysis of the expression pattern of *ApTK* in different tissues. Different letters indicate significant differences in expression levels among different tissues, while the same letter indicates no significant difference in expression levels among different tissues. The full forms of the abbreviations in (**C**,**D**) are as follows: BR, brain; ED, epidermis; SG, silk gland; HE, hemolymph; MT, Malpighian tubule; MD, midgut; FB, fat body. (**E**) Expression analysis of *ApTK* post-starvation in *A. pernyi* larvae. (**F**) Expression analysis of *ApTK* after refeeding treatment in the pre-starved larvae. The asterisks in (**E**,**F**) indicate a significant difference between the control and treatment groups based on ANOVA (* *p* < 0.05; ns, not significant).

The full-length cDNA sequence of *ApTK* was obtained. Sequence analysis showed that the ORF sequence length of *ApTK* was 876 bp, encoding 291 amino acid residues with the cleavage site of the signal peptide between 21 aa and 22 aa (Figure 1A). The sequence of *ApTK* was submitted to GenBank under accession number PX849510. Four typical motifs comprising FMGVR, FYGVR, FIGVR, and FFGMR were observed at the C-terminus (Figure 1A), corresponding to the specific characteristic of insect TKs with an FX_1_GX_2_R structure. The results of amino acid multiple sequence alignment indicated that ApTK, BmTK, and MsTK were highly conserved in their sequence and FX_1_GX_2_R motifs (Figure 1A). Besides this, 23 potential phosphorylation sites (11 on serine, four on threonine, and eight on tyrosine) within ApTK were predicted. The results of domain prediction showed that no conserved domains were found while several intrinsically disordered regions (22 aa-36 aa, 168 aa-225 aa, and 236 aa-251 aa) were present within ApTK. Phylogenetic analysis was conducted for ApTK and TKs from other lepidopteran species. The results indicated that ApTK was clustered on the same branch with TKs from *Manduca sexta*, *B. mori*, and *Ostrinia furnacalis* (Figure 1B).

The results of RT-PCR and qRT-PCR showed that the highest expression of *ApTK* was observed in the midgut, followed by the brain. The expression of *ApTK* was almost undetectable in the other tissues, including the epidermis, hemolymph, fat body, silk gland, and Malpighian tubules (Figure 1C,D).

### 3.2. Differential Expression of ApTK in A. pernyi Larvae After Starvation–Refeeding Treatment

Changes in the gene expression of *ApTK* in *A*. *pernyi* larvae post-starvation–refeeding treatment were examined. The qRT-PCR results showed that *ApTK* was significantly up-regulated at 48 h and reached its highest level at 72 h after starvation compared to the control group (Figure 1E). During refeeding after food deprivation, the expression level of *ApTK* showed a steady decreasing trend, then approached its original level at 72 h post-refeeding (Figure 1F).

### 3.3. Effect of RNAi Targeting ApTK on Body Weight and Energy Metabolism of A. pernyi Larvae

**Figure 2:**
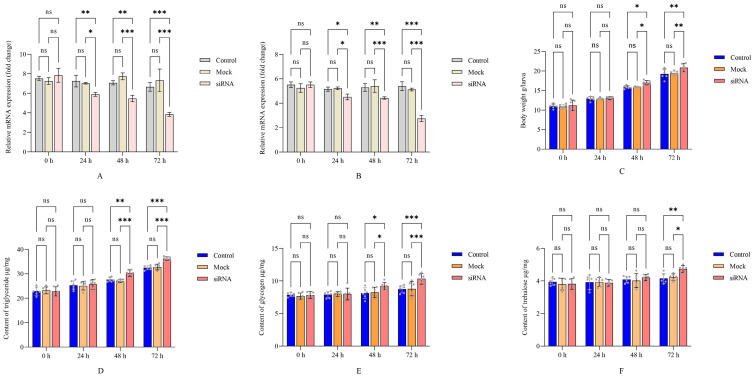
Statistical analysis of larval body weight and energy metabolism after *ApTK* silencing in *A. pernyi*. (**A**) Detection of silencing efficiency of siRNA-mediated RNAi on expression of *ApTK* in midgut of *A. pernyi*. (**B**) Detection of silencing efficiency of siRNA-mediated RNAi on expression of *ApTK* in brain of *A. pernyi*. (**C**) Statistics of larval body weight of *A*. *pernyi* post-RNAi. Energy metabolism, including triglyceride (**D**), glycogen (**E**), and trehalose (**F**), was detected in *A*. *pernyi* larvae after *ApTK* knockdown. Asterisks indicate significant difference between control or mock (DEPC water) and siRNA group based on ANOVA (* *p* < 0.05, ** *p* < 0.01, *** *p* < 0.001; ns, not significant).

The interference efficiency of siRNA targeting *ApTK* in the midgut and brain of *A. pernyi* was first detected by qRT-PCR. The results indicated that the expression of *ApTK* decreased from 24 h and reached the lowest level at 72 h post-siRNA injection as compared to the control and mock groups (Figure 2A,B). Then the larval body weight was measured. The results implied that the body weights of the larvae in the three groups all continued to increase until 72 h. However, the larval body weight of the siRNA group was significantly higher than that in the other two groups starting from 48 h after siRNA injection (Figure 2C).

To verify the correlation between ApTK and energy metabolism, the contents of triglyceride, glycogen, and trehalose in *A*. *pernyi* larvae after silencing of ApTK were detected. The results showed that although the content of triglyceride increased gradually in all groups, significant differences were observed between the siRNA group and both the control and mock groups at 48 h and 72 h (Figure 2D). The content of glycogen also increased more remarkably after *ApTK* silencing than in the control and mock groups, being significantly higher in the siRNA group compared to the other two groups starting from 48 h (Figure 2E), and significant differences in the trehalose content between the siRNA group and the control and mock groups were exhibited at 72 h post-RNAi (Figure 2F).

## 4. Discussion

TKs are highly conserved in invertebrates, with most containing the conserved pentapeptide structure (FX_1_GX_2_R; X_1_ and X_2_ represent variable amino acid residues). The first insect TK was isolated from the locust [25]. This study identified the TK gene (*ApTK*) in the Chinese oak silkworm, *A. pernyi*, and bioinformatic analysis revealed that ApTK contains four typical pentapeptide motifs (FMGVR, FYGVR, FIGVR, FFGMR) at its C-terminus (Figure 1A), consistent with the conserved features of insect TKs [26]. Twenty-three potential phosphorylation sites were observed within ApTK. Among these, Ser/Thr sites near the mature peptide region may regulate precursor processing efficiency or secretion, while C-terminal Tyr sites could influence receptor-binding affinity or signal transduction. Furthermore, comparative analysis with homologous TK sequences from *B. mori* and *M. sexta* showed conservation of cleavage sites (such as Lys/Arg, KR) and phosphorylation patterns (Figure 1A), supporting the functional conservation of this neuropeptide in lepidopteran insects. The predicted potential protein–protein interaction regions (IDRs) may confer conformational plasticity to ApTK, enabling interactions with different receptor subtypes or auxiliary proteins, implying multiple roles in regulating feeding behavior and midgut metabolism. In addition, the close phylogenetic relationship between ApTK and TKs from *B. mori* and *M. sexta* (Figure 1B) suggests that it may be a functional ortholog, although experimental confirmation is required.

Insect TKs were initially discovered in brain tissue and later detected in the midgut, hence often being referred to as “brain–gut peptides” [27]. In *D. melanogaster*, the neurons secreting TKs are mainly distributed in the brain and gut, followed by the ventral nerve cord [8,28]. In *M. sexta*, TK neurons are mainly located in the central nervous system, including the brain and ventral nerve cord, while also occurring in the midgut [29]. Besides these, a TK-like peptide referred to as natalisin was also proved to be expressed in the brain neurons of *D. melanogaster*, *Tribolium castaneum*, and *B. mori* [30]. Our study showed that *ApTK* exhibited different expression patterns in varying tissues of *A. pernyi*, with the highest transcript levels in the brain and midgut. This aligns with findings in the aforementioned insect species and is consistent with the dual roles of TKs as neuromodulators and gut regulatory peptides for nutrient sensing, feeding regulation, and neuroendocrine control [31]. In insects, TKs typically signal through G protein-coupled receptors (GPCRs) belonging to the tachykinin receptor (TKR) family [32,33]. Given that ApTK was highly expressed in the brain and midgut in our study, we infer that ApTK may regulate feeding motivation and behavior by acting on neuronal networks expressing TKR in the brain and directly modulate nutrient absorption, enzymatic activity, and energy substrate metabolism by acting on TKR expressed in the midgut and other metabolic organs.

Insect TKs have been shown to play crucial roles in regulating feeding and energy metabolism [34]. In this study, starvation treatment significantly upregulated the expression of *ApTK* in *A. pernyi* larvae, and it returned to baseline levels after refeeding (Figure 1E,F). This dynamic response indicates that ApTK may act as a hunger signal or as part of the homeostatic response to energy deficit, similar to observations in *B. mori* [13] and *D. melanogaster* [5]. In *A. pernyi*, this up-regulation is likely to prepare the insect for compensatory feeding and metabolic adjustment, highlighting an evolutionarily conserved role for TKs in nutrient sensing and foraging motivation.

To further validate the function of ApTK, we silenced the *ApTK* gene using RNAi. Effective silencing of *ApTK* led to a significant increase in the larval body weight compared to controls (Figure 2C), which indicates a potential anorexigenic (appetite-suppressing) role for *ApTK* in *A. pernyi*. Our results match findings in *B. mori*, where disruption of TK signaling leads to increases in food intake and body mass [13], suggesting that TK may serve as a critical factor in regulating feeding in lepidopteran insects.

Lowered glycemia is an important trigger for hunger and food-seeking behavior in insects [35,36]. Upon starvation, glycogen and trehalose are broken down to sustain basal energy homeostasis, while the stronger hunger signal leads to the metabolism of lipids, especially triglyceride [37]. Starvation stress also activates a series of neuropeptide signaling regulatory processes to maintain lower physiological metabolic activity [38]. Our metabolic assays showed that silencing *ApTK* caused a significant accumulation of triglyceride, glycogen, and trehalose in *A. pernyi* larvae (Figure 2D–F), indicating that ApTK signaling may serve as a negative regulator of energy storage. Downregulation of ApTK likely triggers hunger signaling in *A. pernyi*, which may lead to increased food intake and consequently elevate the contents of these energy reserves. In *Drosophila*, TK signaling interacts with insulin signaling to regulate carbohydrate and lipid metabolism; its disruption of TK causes metabolic imbalance and fat accumulation [11,12]. The increased levels of the three energy metabolites in *A. pernyi* suggest that ApTK may exert an inhibitory effect on insulin signaling or other anabolic hormonal pathways. Elevated trehalose, a major blood sugar in insects, also hints at a role for ApTK in regulating glucose homeostasis.

However, TK functions exhibit diversity and species-specificity across different insect groups (Table 1). We systematically compared the function of ApTK in *A. pernyi* with that of TKs in other representative insects. Among lepidopteran insects, the function of BmTK in *B. mori* is most similar to that of ApTK, as both negatively regulate feeding and body weight [13]. The coordinated regulation of multiple energy storage substances by ApTK may hold particular adaptive significance for wild *A. pernyi* coping with intermittent food intake, while TK in *M. sexta* is more focused on regulating local digestive processes such as midgut peristalsis [31]. For non-lepidopteran insects, TK functional networks are more diverse. For instance, DTK in *D. melanogaster* is deeply integrated into the insulin metabolic network, regulating stress responses [11,12]; TK in *R. prolixus* primarily regulates muscle contractions associated with liquid feeding [14]; and TK in *P. americana* influences digestive enzyme activity [5,15]. In summary, compared to TKs in other reported insects, the regulatory characteristics of ApTK in *A. pernyi* are: (1) sensitivity to starvation, with sustained upregulation of expression; (2) acting as a negative regulatory switch for feeding behavior; and (3) exerting a coordinated inhibitory effect on overall energy storage homeostasis.

It should be noted that the present study primarily focused on the functional characterization of ApTK during the larval stage of *A. pernyi*. While our results demonstrate its critical role in larval growth and development, the potential involvement of ApTK in adult reproduction, diapause regulation, or other physiological processes remains unexplored. Future investigations employing stage-specific knockdown or conditional knockout techniques in adult moths could elucidate these aspects, thereby providing a more comprehensive understanding of ApTK’s multifunctional roles across the insect life cycle. From an applied perspective, *A. pernyi* holds significant economic value as a silk-producing insect and a source of edible products. Our findings on ApTK’s regulatory effect on larval growth suggest its potential as a molecular target for optimizing rearing strategies. For instance, precise modulation of *ApTK* expression—via dietary supplements, RNAi-based approaches, or genetic selection—could enable better control of larval weight and developmental timing. Such targeted interventions may contribute to more efficient and sustainable sericulture practices.

In conclusion, the results of this study indicate that the TK signaling system in *A. pernyi* is a critical regulator at the nexus of feeding behavior and energy metabolism. ApTK responded to starvation stress, and our knockdown results suggest a function in restraining excessive weight gain and energy storage, implying that this neuropeptide may represent one of the potential mediators involved in maintaining metabolic balance in *A. pernyi*. However, it is important to note a limitation of the current study. While our *ApTK* knockdown experiments suggest a role in restraining energy storage, definitive proof of this function would require complementary gain-of-function approaches, such as peptide injection or genetic overexpression, to observe the opposite phenotypic effects. Future studies employing these strategies will be crucial to conclusively establish ApTK as a key mediator of metabolic balance in *A. pernyi*. Overall, our findings identify ApTK as a key endogenous regulator of larval growth and energy homeostasis in *A. pernyi*. This provides a potential molecular target for the future development of novel strategies to manage growth and metabolic health in this economically important insect and possibly in related lepidopteran species.

## Figures and Tables

**Table 1:** The expression sites, regulatory phenotypes, and signaling pathways of TK in different species.

Species (Order)	Gene/Peptide Name	Major Expression Sites	Key Physiological Functions/ Phenotypes	Involved Putative or Known Signaling Pathways	References
*A. pernyi* (Lepidoptera)	ApTK	Brain; midgut	Increases body weight; elevates triglyceride, glycogen, and trehalose levels.	Likely acts via the brain–gut axis to exert anorexigenic (feeding-inhibitory) and energy storage-limiting effects; may interact with metabolic hormone pathways like insulin.	This study
*B. mori* (Lepidoptera)	BmTK	Brain; midgut; subesophageal ganglion	Upregulated by starvation; knockdown increases food intake and body weight; overexpression shortens food-searching time after starvation.	Regulates feeding motivation and satiety; influences foraging behavior.	[13]
*M. sexta* (Lepidoptera)	Mas-TK I-VII	Central nervous system; midgut; hindgut	Stimulates midgut and hindgut muscle contractions; involved in regulating peristalsis of the digestive tract.	Acts as a neuromuscular modulator on smooth muscle.	[31]
*D. melanogaster* (Diptera)	DTK	Brain; gut, ventral nerve cord	Regulates insulin secretion; affects trehalose levels, starvation resistance, oxidative stress; involved in olfactory modulation.	Interacts with insulin signaling pathway; regulates metabolic homeostasis and stress response.	[5,10,11,12]
*R. prolixus* (Hemiptera)	Rhopr-TK	Brain; midgut; hindgut; salivary glands	Increases peristaltic frequency and amplitude in salivary glands and hindgut.	Regulates fluid transport and muscle activity associated with feeding (blood ingestion) and excretion.	[14]
*P. americana* (Blattodea)	Pea-TK	Midgut endocrine cells	Upregulated by nutrients; affects activities of digestive enzymes; regulates intestinal lipid metabolism.	Acts as a “brain–gut peptide” to locally regulate digestion and absorption processes.	[5,15]

## Data Availability

The original contributions presented in this study are included in the article/Supplementary Material. Further inquiries can be directed to the corresponding author.

## Supplementary Materials

The following supporting information can be downloaded at: https://www.mdpi.com/article/10.3390/insects17030257/s1, Table S1: Primer sequences used in this study. Table S2: The detailed information of tachykinins from the Lepidoptera insects used for amino acid sequence alignment and phylogenetic tree construction.
